# Inborn Errors of Immunity in Children with Autoimmune and Allergic Complaints: A Single Center Experience from Diagnosis to Treatment

**DOI:** 10.3390/biomedicines11051299

**Published:** 2023-04-27

**Authors:** Valentina Boz, Alessandra Tesser, Martina Girardelli, Francesca Burlo, Alessia Pin, Giovanni Maria Severini, Ginevra De Marchi, Federico Verzegnassi, Samuele Naviglio, Alberto Tommasini, Erica Valencic

**Affiliations:** 1 Department of Pediatrics, Institute for Maternal and Child Health IRCCS Burlo Garofolo, Via dell’Istria 65, 34137 Trieste, Italy; 2 Department of Medical, Surgical and Health Sciences, University of Trieste, 34137 Trieste, Italy; 3 Rheumatology Clinic, Department of Medical and Biological Sciences, Azienda Sanitaria Universitaria Friuli Centrale c/o, University of Udine, 33100 Udine, Italy

**Keywords:** autoimmunity, allergy, immunodysregulation, inborn errors of immunity, precision treatments, primary immune deficiency

## Abstract

Inborn errors of immunity (IEI) associated with immune dysregulation are not sufficiently addressed in shared recommendation, resulting in delayed diagnosis and high morbidity. The availability of precision medicine for some of these immune defects makes it urgent to evaluate effective strategies to diagnose and treat such defects before the occurrence of severe complications. A diagnosis of an IEI in these patients enabled the use of a more specific treatment in most cases, and these have the potential to prevent further disease progression. We studied immune dysregulation diseases in 30 patients with autoimmune or allergic phenotypes, exploiting data from clinics and immunophenotype, genetic and transcriptome investigations, and 6 of them were diagnosed with a monogenic disorder. Our results confirm that a non-negligible number of children with IEIs may present with signs and symptoms of immune dysregulation and share many features with common multifactorial immune conditions. Reaching a genetic diagnosis becomes more likely in the presence of multiple clinical manifestations, especially when in association with abnormalities of lymphocytes subsets and/or immunoglobulins levels. Moreover, 5 of 6 patients that obtained a diagnosis of monogenic disorder received precision therapy, in four cases with a good or moderate response.

## 1. Introduction

The last two decades have witnessed a rising awareness that primary immune defects may present with signs and symptoms due to immune dysregulation, even in the absence of infections [1,2]. Accordingly, the term Inborn Errors of Immunity (IEI) is increasingly being used instead of primary immune deficiency (PID), aiming to comprise a wider spectrum of clinical presentations. However, including inflammation, autoimmunity, allergy and lymphoproliferation among clinical features supportive of an IEI makes the diagnostic process even more challenging. In fact, even though typical combinations of signs and symptoms of immune dysregulation can be a clue for an IEI, the whole clinical phenotype often builds up over time and may not be immediately apparent. Furthermore, since many of these complaints can also be found in patients with sporadic multifactorial conditions, patient selection for further immunologic and genetic analysis is challenging, as there is a high risk of a low yield of the tests or of missing the few patients with genuine IEIs. A timely diagnosis of an underlying IEI is desirable, not least because it may enable the use of target treatments in some patients, possibly preventing worsening clinical conditions. To this purpose, shared protocols are needed to improve patient selection among those presenting with immune dysregulation phenotypes to reach more effective selection criteria [3,4]. Some “red flags” have been proposed based on clinical experience, including multi-organ autoimmune/inflammatory involvement, lymphoproliferative manifestations, presentation in early childhood and refractoriness to conventional therapies [5]; nevertheless, patient selection for further immunological and genetic work-up is still largely at the physician’s discretion.

The aim of this study was to explore the feasibility of defining a priori criteria for selecting patients who may deserve a deep immunological and genomic study to detect immune dysregulation disorders and to assess the therapeutic impact of a definite diagnosis. Indeed, the well-established warning signs proposed by the Jeffrey Modell Foundation (JMF) to raise the suspicion of a primary immunodeficiency are mostly related to the risk of infections and may not help addressing the possibility of an immune dysregulation disease if it is not associated with serious or recurrent infections. To consider autoimmunity, allergy or lymphoproliferation in addition to the 10 JMF warning signs may enable the encompassing of a wider range of IEIs, but it also risks widening the study population too much. For this reason, we combined clinical signs of disease with immune disturbance (hypogammaglobulinemia or hypergammaglobulinemia, leukopenia, eosinophilia and lymphoproliferation) to improve the detection of subjects with IEIs. We report the results of our experience in the immunologic and genetic evaluation of children presenting with multiple clinical manifestations of immune dysregulation. Within this series, we recorded which clinical or immunologic features impacted the final diagnosis of an IEI. Moreover, we described cases in which the diagnosis affected the treatment, enabling the use of drugs targeted to the underlying pathogenesis mechanisms of the disease.

## 2. Materials and Methods

### 2.1. Recruitment Criteria

Within a research project, we enrolled subjects meeting at least one of the following set of criteria, which we considered possibly supportive of an immune dysregulation:

1. One or more autoimmune disorders associated with at least one of the following: hypergammaglobulinemia or hypogammaglobulinemia, hypereosinophilia (>1500/mcL) or chronic lymphoproliferation (lymphadenopathy and/or splenomegaly);
2. Severe dermatitis plus hyper-IgE (>2000 UI/mL) and at least one of the following: recurrent infections, skeletal and joint disorders, recurrent pneumonia or pyogenic cutaneous abscesses;
3. Idiopathic hypereosinophilia (>5000/mcL) plus hypergammaglobulinemia (values above the 97.5% for age and sex for at least one among IgG, IgA and IgM) and at least one of dermatitis, enteropathy and chronic lymphoproliferation;
4. Severe dermatitis refractory to topical glucocorticoid treatment; dermatitis is considered severe if it affects at least 10 percent of the body surface and it affects quality of life and disrupts sleep, despite continuous topical treatment with glucocorticoid-based ointments;
5. Severe food anaphylaxis refractory to oral desensitizing treatments. At our hospital, oral desensitization is proposed to all patients who experienced episodes of anaphylaxis to foods for which it is not easy to avoid the risk of allergic reactions for accidental exposures, such as for cow’s milk and egg proteins and that contain high levels of food-specific IgE antibodies.

Symptoms and signs were intentionally broad to encompass a relatively large spectrum of clinical presentations.

Patients in group 1 were defined, only for purposes of the present study, as having a predominantly “autoimmune” phenotype, while the others from group 2 to group 5 were broadly defined as having an “allergic” phenotype. The study was approved by the local Ethics Committee (CEUR-2020-PR-13 approval code, on date 15 September 2020). We enrolled consecutive patients meeting inclusion criteria from January 2018 to December 2021. After obtaining patients/caregivers’ informed consent, we collected DNA, RNA and peripheral blood mononuclear cells from each patient, and recorded clinical and laboratory data on a structured database.

### 2.2. Flow Cytometry Analysis

#### 2.2.1. Immunophenotype

For most patients, we performed immunophenotyping with an extended cytometry panel in order to evaluate various lymphocyte subpopulations. Among B cell subpopulations we distinguished naïve follicular B cells (B naive), defined as CD45^++^CD19^+^IgD/IgM^+^CD27^−^; IgM B memory cells (B mem), defined as CD45^++^CD19^+^IgD/IgM^+^CD27^+^; switched memory B cells (B swi), defined as CD45^++^CD19^+^IgD/IgM^-^CD27^+^ and transitional B cells/recent bone marrow emigrants (RBE), defined as CD45^++^CD19^+^IgD/IgM^+^CD10^+^. Among T cells, we examined recent thymic emigrants (RTE), defined as CD45^++^CD3^+^CD4^+^CD31^+^CD45RA^+^; double negative T cells (DNT), defined as CD45^++^CD3^+^CD4^-^CD8^-^TCR a/b^+^; CD4/CD8 ratio; senescent CD8 T cells, defined as CD45^++^CD3^+^CD8^+^CD57^+^CD45RA^+^ and activated T cells, defined as CD45^++^CD3^+^CD4^+^CD69^+^ and as CD45^++^CD3^+^CD4^+^HLA^-^DR^+^.

#### 2.2.2. CTLA4 Expression

Peripheral blood mononuclear cells (PBMCs) were left unstimulated or were treated with PMA and ionomycin for 2 h or with PHA for 24 h. Cells were then stained with anti-CD3 VioBlue, anti-CD4 APC and anti-CTLA4 PE antibodies to analyze CTLA4 expression in CD4^+^ T cells and in resting lymphocytes with flow cytometry.

#### 2.2.3. LRBA Expression

PBMCs were left unstimulated or were treated with PHA and incubated for 72 h at 37 °C, 5% CO_2_. Then, cells were stained with surface antibodies CD3 VioBlue, CD4 APC and CD69 PE-Vio770. Finally, cells were fixed and permeabilized and then marked with anti-LRBA antibody for 30 min and then with secondary antibody for another 30 min to analyze LRBA expression in CD3^+^CD69^+^ T cells and in resting lymphocytes with flow cytometry.

#### 2.2.4. STAT1 Expression

PBMCs were left unstimulated or were treated with IFN-alpha2a. After 24 h of incubation at 5% CO_2_ at 37 °C, cells were stained with antibodies to cell surface antigens CD45 V500-C, CD3 VioBlue, CD4 PE and then fixed and permeabilized. PBMCs were finally stained with anti-STAT1 antibody or with an isotype control antibody. The expression level of STAT1 was analyzed in CD4^+^ T cells with flow cytometry.

#### 2.2.5. S6 Phosphorylation

PBMCs were left unstimulated or were treated with Dynabeads Human T-activator CD3/CD28 and incubated for 4 h at 37 °C, 5% CO_2_. Cells were surface-stained with CD4 APC and CD3 VioBlue and then fixed and permeabilized with paraformaldehyde 4% and methanol 90%, respectively, to stain with anti-pS6 PE or corresponding isotype PE. The level of S6 phosphorylation was analyzed in CD4^+^ T cells activated in flow cytometry.

For all analyses conducted in flow cytometry, samples were acquired with MACSQuant Analyzer 10 cytometer and analyzed with FlowLogic 7.2.1 software.

### 2.3. Genetic Analysis

Genetic analysis by target gene panels (gene described in Table S1: Target gene panels involved in genetic analysis) or whole exome sequencing (WES) was performed in selected patients. WES analysis was conducted by an external service, Biodiversa (Rovereto, Italy), through Next Generation Sequencing (NGS) on the Illumina platform. Variants considered to be causatively correlated to the clinical phenotype were confirmed through Sanger sequencing and family segregation analysis. Whenever feasible, we further tested the pathogenicity of the detected mutations with appropriate functional assays.

### 2.4. RNA Sequencing (RNAseq) Analysis

Transcriptomic analysis was performed on peripheral whole blood cells via RNA sequencing. A differential gene expression pipeline was run in comparison with a group of young healthy subjects. Principal component analysis (PCA), useful for data visualization, was conducted with DESeq2 (v. 1.32.0) [6] to define and display the gene expression overall variability between subjects considering the 1000 most variable genes across all the samples detected according to the specific function intrinsic to this R package, removing differences due to gender and sequencing batches using the function removeBatchEffect of the package limma (v. 3.50.3) [7]. The most representative genes were selected according to a fold change greater than 2-fold increase/decrease and adjusted *p*-value < 0.05.

## 3. Results and Discussion

Overall, 30 patients were enrolled; 14 patients met the “autoimmune phenotype” criteria, 13 met the “allergic phenotype” criteria and 3 showed an overlapping presentation. For most patients, we performed immunophenotyping with an extended cytometry panel. Target gene panels or whole exome sequencing were also analyzed in 22 selected patients (Table S2: Summary table of performed analysis of all 30 enrolled patients). RNA sequencing (RNAseq) analysis was also performed, both to explore transcriptomic correlates of genetic defects and to search for shared pathways between patients with similar phenotypes (Table S2: Summary table of performed analysis of all 30 enrolled patients).

Genetic investigations reached a definitive genetic diagnosis in six cases (Table 1). LPS responsive beige-like anchor protein (LRBA) deficiency and cytotoxic T-Lymphocyte Antigen 4 (CTLA4) haploinsufficiency were diagnosed, respectively, in one and two patients, all presenting with the autoimmune phenotype, and for all of them, the diagnosis was confirmed via LRBA and CTLA4 expression with flow cytometry. Immunodysregulation polyendocrinopathy enteropathy X-linked syndrome (IPEX) due to mutation in the *FOXP3* gene was found in one patient with a predominantly allergic phenotype. Mutations in *STAT1*, and the *TNFRSF13B* (TNF receptor superfamily member 13B) gene, respectively, associated with Signal Transducer and Activator of Transcription 1 (STAT1) gain-of-function (GOF) syndrome, as well as Common Variable Immune Deficiency (CVID), were found in one patient each; both patients met both the autoimmune and the allergic phenotype criteria. STAT1 expression was also studied with flow cytometry to functionally confirm the diagnosis in the patient with STAT1 GOF syndrome (Table 1). 

**Figure 1:**
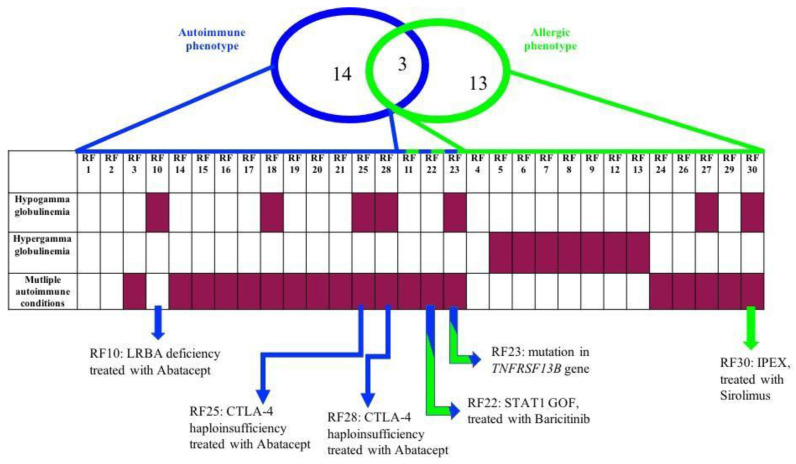
Enrolled patients according to autoimmune phenotype (criteria 1) and allergic phenotype (criteria 2, 3, 4 and 5) who present laboratory anomalies and/or multiple autoimmune conditions (purple rectangle) or do not (white rectangle). Patients with genetic diagnoses for each group and associated treatment are indicated at the end of the figure. RF is a code to anonymize patients with progressive numbers based on the timing of enrollment.

A correlation with presenting features showed that patients in whom a genetic diagnosis was reached tended to present with hypogammaglobulinemia more frequently than those with negative genetic results (Figure 1). In particular, considering the presence of hypogammaglobulinemia and multiple (more than one) autoimmune conditions, a genetic diagnosis was made in 5/7 patients with both hypogammaglobulinemia and multiple autoimmune conditions, 6/19 patients with multiple autoimmune conditions, 5/7 patients with hypogammaglobulinemia and 0/2 patients with neither hypogammaglobulinemia nor multiple autoimmune conditions. The small numbers did not allow for statistical analysis.

Of 29 subjects who underwent extensive immunophenotyping, 16 had a significant alteration of T and/or B lymphocyte subsets (Table S3: Summary table of obtained results of all 30 enrolled patients). Notably, a history of frequent or unusual infections was present in three of six patients with a genetic diagnosis (Table S3: Summary table of obtained results of all 30 enrolled patients).

Overall, a definite diagnosis of IEI was possible only in two of six subjects on the basis of genetic results alone, while in the remaining four cases, only a deep immunological assessment allowed a definite interpretation of genetic results, providing supportive phenotypic or functional data to classify the variants of unknown significance according to the American College of Medical Genetics and Genomics (ACMG) standards [10].

Genetic diagnosis impacted therapeutic choices in some cases, allowing for the choice of a molecularly targeted treatment (Table 1). In particular, three patients received treatment with abatacept, which has already been demonstrated to be useful for LRBA deficiency and CTLA4 haploinsufficiency in cases wherein hematopoietic stem cell transplantation is not indicated or feasible [11]. In one patient, a boy with refractory autoimmune gastritis and lymphoproliferative complaints, the response to treatment was dramatic (clinical case described elsewhere [12]). Abatacept was administered at a very low dosage (250 mg monthly, equal to 7 mg/kg), which was considerably effective in improving either the symptoms or the endoscopic status. The second case consisted of a woman with CTLA4 haploinsufficiency. She also presented a long-lasting story of hematological, gastroenterological and respiratory complaints and an autoimmune clinical phenotype resembling Systemic Lupus Erythematosus After starting abatacept, she experienced a partial improvement in cytopenia and enteric symptoms. Unfortunately, just one year after starting abatacept, she was diagnosed with gastric cancer and had to suspend the immunosuppressive treatment during the cancer treatment. The third and final patient treated with abatacept was another woman with CTLA4 deficiency and a long history of hematological autoimmunity since her adolescence, who also developed inflammatory bowel disease-like inflammation and severe respiratory infections with interstitial pneumonia and bronchiectasis with the need for respiratory and nutritional support. Unfortunately, she was considered ineligible for hematopoietic stem cell transplantation and/or lung transplantation. Abatacept was well-tolerated, but allowed for only a mild reduction of glucocorticoids dosage, which was not enough to change the course of her disease, with death following two years after the start of treatment. Recent case reports described the effective use of abatacept to treat pediatric patients with either autoimmune or autoinflammatory manifestations and an underlying CTLA4 deficiency [13,14,15]. Another case, a girl with autoimmune thyroiditis, gastritis, oral ulcerations and mucosal candidiasis, with positive antinuclear and anti-DNA antibodies, was treated with hydroxychloroquine based on a diagnosis of Systemic Lupus Erythematosus, with scarce control of symptoms. Only after the diagnosis of STAT1 gain-of-function syndrome could a treatment with a JAK inhibitor be prescribed, resulting in a dramatic improvement in control of symptoms, as described in the literature [16,17]. The last case in which a molecular diagnosis impacted treatment was a boy diagnosed with IPEX syndrome. The diagnosis resulted in the mechanistic use of sirolimus, which led to an improved control of enteropathy and growth, as a bridge to hematopoietic stem cell transplantation. A dosage of 0.08 mg/kg daily was effective in controlling the disease, with good hematic drug levels. This dosage was considerably lower than that already reported [18].

**Figure 2:**
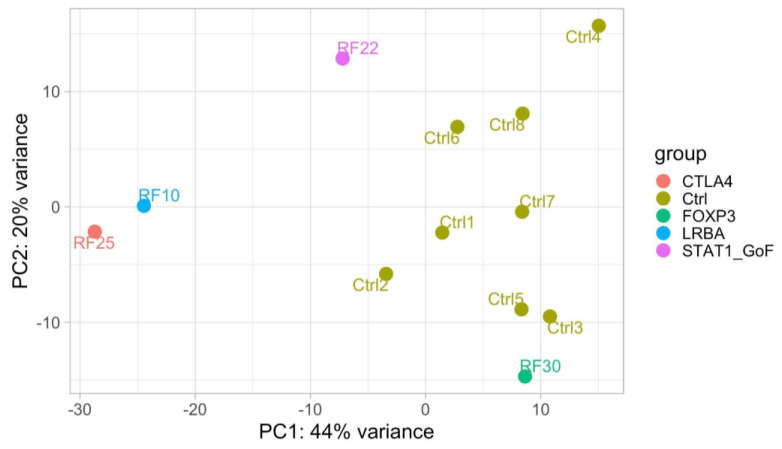
Distribution of patients and healthy controls (Ctrl, code to anonymize patients with progressive numbers to distinguish one from the others) according to the 1000 most variable genes, normalized for gender and batches. RF, code to anonymize patients with progressive numbers based on the timing of enrollment. Figure obtained by DESeq2 (v. 1.32.0).

We also investigated gene expression in peripheral blood cells from subjects with confirmed monogenic disorders to search for profiles associated with specific genotypes or phenotypes. The bioinformatic analysis was carried out for each patient on the 1000 most variable genes compared with healthy controls. Figure 2 shows the results of principal component analysis (PCA) to highlight overall differences and similarities between patients with distinct monogenic disorders and controls (patients RF28 and RF23 were not included as their RNA was not available). PCA is a statistical method allowing to render the complexity of a large set of variables on a bidimensional chart.

In this analysis, samples tend to cluster near each other when they share a similar gene expression profile. Patients tended to cluster separately from healthy controls, with the exception of the patient with IPEX, who, however, still showed a mild expression of the disease. Notably, the patient with LRBA deficiency clustered close to the patient with CTLA4 haploinsufficiency, as expected, considering the involvement of shared pathways in the two diseases. Unfortunately, a comparison with patients with negative genetic results was not performed due to the low number of available RNA samples from this group. Thus, we cannot assess whether monogenic-disease-specific-like profiles may impact the classification of diseases for therapeutic purposes in individuals with negative genetic results.

Overall, our data confirm that a non-negligible number of subjects with IEIs may present with signs and symptoms of immune dysregulation, often without a history of infections. While these patients share many features with common multifactorial immune conditions arising in the general population, reaching a genetic diagnosis becomes more likely in the presence of multiple clinical manifestations, especially when in association with abnormalities of lymphocyte subsets and/or hypogammaglobulinemia. In fact, this latter sign seemed to represent a strong predictor of a genetic diagnosis of IEI.

Thus, recommendations to improve an early diagnosis of IEIs might be developed by weighting the number of immune complaints, the age at their onset and the presence of immune abnormalities. Of note, several laboratory anomalies in immune parameters appear worthy of being considered as supportive for a diagnosis of IEI, in particular, hypogammaglobulinemia and lymphocyte subset anomalies. Furthermore, the assessment of immunophenotype must not be limited to the main lymphocyte subsets, but should include specific subsets depending on the clinical picture.

One characteristic shared by several patients in our series concerned their referrals to multiple specialties, because of complex clinical pictures encompassing multiple organ involvement. Even if comorbidities are not unusual in adults, they are much more rare the younger the subjects are. For this reason, the multi-organ involvement of an immune disease in children and young adults should prompt the suspicion of a dysregulatory PID and should warrant proper immunological and genetic investigations. This is even more important as some dysregulatory IEI can have precision treatments that can prevent a worse disease progression.

Another issue raised by our experience concerns the importance of referring subjects with a suspected IEI to specialized centers, where different diagnostic approaches can be integrated, for example, to determine the significance of unclear genomic analysis with functional immune assays.

A limitation of our study relies on its monocentric nature. This may represent a limitation both for the limited number of patients identified and for the possible existence of selection bias due to the main areas of expertise at our institute. However, this is also an added value for our study, since it describes a real-life experience of diagnosis and care of rare diseases in the field of immune defects. In fact, the study may be the starting point of novel multicenter research to explore the potential of multi-omic analysis in the field of monogenic and multifactorial immune dysregulation disorders.

## 4. Conclusions

A diagnosis of an IEI in our study allowed for the use of a more specific treatment in most cases. This is of particular importance since more effective treatments have the potential to prevent further disease progression [3,19]. Our experience supports recent suggestions to integrate general recommendations for suspecting an IEI (e.g., the 10 warning signs from Jeffrey Modell Foundation) by including signs of immune dysregulation [2]. We acknowledge that our study, being monocentric, may be limited due to reference bias and that the selection criteria used in our research cannot be proposed for general use in clinical practice. Nevertheless, they can serve to highlight the importance of some clinical and laboratory features for the suspicion of an IEI. Notably, our study also shows that despite the great innovation brought by NGS techniques, genetic evaluation still needs to be combined with immunological and clinical data in most cases to reach a definite diagnosis [20].

## Figures and Tables

**Table 1:** Summary table of performed analysis and results of patients with a defined genetic diagnosis.

Pt	Age at Onset (years)	Clinical Features	Mutations	Inheritance	Segregation Analysis	Immune Phenotype and Immunoglobulins	Functional Assays	Transcriptome Analysis	Targeted Treatment
RF10	2	autoimmune gastritis; splenomegaly; multiple lymphadenopathy; hypogammaglobulinemia; anemia	*LRBA* c.C6415T p.R2139X; c.C7315T p.R2439X	AR	*LRBA* c.C6415T p.R2139X (father) c.C7315T p.R2439X (mother)	DNT αβ↑; IgA↓	LRBA, CTLA4 and pS6 expression	LRBA ↓	Abatacept
RF25	15	recurrent fevers; autoimmune thrombocytopenia; autoimmune hemolytic anemia; leukopenia; recurrent infections; severe hypogammaglobulinemia	*CTLA4* c.G160A p.A54T	AD	*CTLA4* c.G160A p.A54T (mother and brother); father, daughter and maternal aunt wild-type	B lymphocytes ↓; RTE↓; IgG↓; IgM↓; IgA↓	CTLA4 and pS6 expression	signaling related to B cells population	Abatacept
RF28	13	autoimmune thrombocytopenia; severe hypogammaglobulinemia; chronic enteropathy; insulin-dependent type 1 diabetes mellitus; autoimmune thyroiditis; frequent infections	*CTLA4* c.C223T p.R75W	AD	nd	B lymphocytes ↓; RTE↓; IgG↓; IgM↓; IgA↓	CTLA4 and pS6 expression	nd	Abatacept
RF22	2	recurrent oral candidiasis; aphthous stomatitis (RAS); autoimmune thyroiditis, autoimmune gastritis; hemolytic anemia	*STAT1* c.A1721C p.N574T	AD	nd	normal	STAT1 expression	√	Baricitinib
RF30	0.5	diarrhea; atopic eczema; leukocytosis; hypereosinophilia; coeliac disease; hypogammaglobulinemia (IgG)	*FOXP3* c.748_750ofAAG p.K250del	X-linked	nd	T lymphocytes CD8^+^↑; IgG↓	nd	√	Sirolimus
RF23	2	autoimmune thyroiditis; autoimmune gastritis; alopecia; polyarthritis; hypogammaglobulinemia; anemia	*TNFRSF13B* c.T310C p.C104R	AD	nd	switched memory B cells↓; IgG↓; IgA↓	nd	nd	-

Note: RF, code to anonymize patients with also progressive number based on the timing of enrollment; AR, autosomal recessive; AD, autosomal dominant; nd, not done; -, not found; √, analysis done; ↓, down expressed; ↑, over expressed; T lymphocytes (CD3^+^); B lymphocytes (CD19+); RTE = Recent Thymic Emigrants (CD3^+^CD4^+^CD31^+^CD45RA^+^); RBE = Recent Bone marrow Emigrants (CD19^+^ CD10^+^ CD38^+^); Regulatory T cells (CD3^+^ CD4^+^ CD25^+^ CD127^−^); DNTαβ = double negative T cells with αβ T cell receptor (CD3^+^ CD4^−^ CD8^−^ TCRαβ^+^); Switched Memory B cells (CD19^+^ CD27^+^ IgD/IgM^−^); CD8 memory T cells. Normal values of immunoglobulins are considered between the 2.5% and the 97.5% for each class. Normal values of lymphocyte subsets are referred and adapted from reference values described in the literature [8,9].

## Data Availability

All data generated or analyzed during this study are available upon request to the corresponding author and will be deposited in publicly available repositories within the next year.

## Supplementary Materials

The following supporting information can be downloaded at https://www.mdpi.com/article/10.3390/biomedicines11051299/s1: Table S1: Target gene panels involved in genetic analysis; Table S2: Summary table of performed analysis of all 30 enrolled patients and Table S3: Summary table of obtained results of all 30 enrolled patients.
