# Supplementary material for: Inborn Errors of Immunity in Children with Autoimmune and Allergic Complaints: A Single Center Experience from Diagnosis to Treatment
Source: Biomedicines. 2023 Apr 27;11(5):1299. doi: 10.3390/biomedicines11051299 (PMC10215820; doi:10.3390/biomedicines11051299)
Supplement: Supplementary file 1 [file biomedicines-11-01299-s001.zip › biomedicines-2283178-supplementary/Table S2.pdf]

Table S2. Summary table of performed analysis of all 30 enrolled patients.

Patient	Genetic analysis	Immuno phenotype	Functional assay	Transcriptomic analysis
RF1	nd	✓	nd	nd
RF2	nd	✓	nd	nd
RF3	WES	✓	STAT3 and STAT5 phosphorylation	nd
RF4	nd	✓	nd	nd
RF5	WES	✓	CD86	nd
RF6	nd	✓	nd	nd
RF7	nd	✓	STAT3 and STAT5 phosphorylation	nd
RF8	nd	✓	nd	nd
RF9	nd	✓	CD86	nd
RF10	Panel of genes	✓	CD86, CTLA4 expression, LRBA expression, S6 phosphorylation	✓
RF11	WES	✓	S6 phosphorylation	✓
RF12	WES	nd	nd	nd
RF13	nd	✓	CD86	nd
RF14	WES	✓	CD86	nd
RF15	Invitae panel	✓	CD86	nd
RF16	WES	✓	STAT3 and STAT5 phosphorylation, FOXP3 expression	nd
RF17	WES	✓	CD86, STAT3 and STAT5 phosphorylation, FOXP3 expression, TNFa expression	✓
RF18	WES	✓	CTLA4 expression, FOXP3 expression	nd
RF19	WES	✓	nd	✓
RF20	WES	✓	CD86, FOXP3 expression	nd

RF21	Invitae panel	✓	CD86, STAT3 phosphorylation	nd
RF22	Panel of genes	✓	STAT1 expression	✓
RF23	Invitae panel	✓	nd	nd
RF24	Panel of genes	✓	nd	nd
RF25	Invitae panel	✓	CD86, CTLA4 expression, S6 phosphorylation	✓
RF26	Panel of genes	✓	nd	nd
RF27	Panel of genes	✓	nd	nd
RF28	Panel of genes	✓	CTLA4 expression, S6 phosphorylation	nd
RF29	Panel of genes	✓	CD86	nd
RF30	WES	✓	nd	✓

Note: RF, code to anonymize patients with also progressive number based on the timing of enrollment; nd, not done; -, not found; ✓, analysis done. CD86 expression: CD86 is the more present isoform in B lymphocytes of CD28 receptor for which abatacept, like CTLA4, competes to inhibit the immune response triggered by the link between CD28 and the receptor CD86. So, we investigated CD86 expression in B lymphocytes cells versus healthy controls in flow cytometry to see if it was upregulated.

S6 phosphorylation: to investigate the link between FAS/FASLG pathway and CTLA4 pathway we analyzed the activation of mTOR/PI3K due to the ability of both pathways to influence the latter.
