# Supplementary material for: Inborn Errors of Immunity in Children with Autoimmune and Allergic Complaints: A Single Center Experience from Diagnosis to Treatment
Source: Biomedicines. 2023 Apr 27;11(5):1299. doi: 10.3390/biomedicines11051299 (PMC10215820; doi:10.3390/biomedicines11051299)
Supplement: Supplementary file 1 [file biomedicines-11-01299-s001.zip › biomedicines-2283178-supplementary/Table S3.pdf]

Table S3: summary table of obtained results of all 30 enrolled patients

Patient	Clinical features	Mutations discovered	Immuno phenotype	Functional assays
RF1	nd	nd	normal	nd
RF2	autoimmune thyroiditis (maternal familiarity); alopecia; maternal familiarity for breast cancer	nd	normal	nd
RF3	trilinear autoimmune cytopenia; urticaria; allergic rhinitis	-	RBE ↑;	pSTAT3 ↓
RF4	severe dermatitis	nd	RBE ↑;	nd
RF5	intractable eczema; hypergammaglobulinemia (IgE)	-	normal	-
RF6	urticaria; angioedema; recurrent pericarditis; hypereosinophilia; unexplained fever; paternal familiarity for Hodgkin's lymphoma; hypergammaglobulinemia (IgM)	nd	T lymphocytes CD8+ ↑;<br>CD4/CD8 ↓;<br>RTE ↓	nd
RF7	severe atopic dermatitis; severe eczema; food allergy; hypereosinophilia; persistent increase in inflammation indices; hypergammaglobulinemia (IgE)	nd	normal	-
RF8	poor severe growth; hypereosinophilia; ESR always high; hypergammaglobulinemia (IgE)	nd	B lymphocytes ↓;<br>T lymphocytes CD8+ ↑;	nd
RF9	bronchospasm at 2 years; hypereosinophilia; chronic cough; hypergammaglobulinemia (IgA)	nd	normal	-
RF10	autoimmune gastritis; splenomegaly; multiple lymphadenopathy; hypogammaglobulinemia; anemia	c.C6415T_p.R2139X<br>c.C7315T_p.R2439X<br>in LRBA gene	DNT αβ↑	LRBA↓;<br>CTLA4↓; pS6↓
RF11	primary hypothyroidism; autoimmune enteropathy; rheumatoid arthritis; trilinear autoimmune cytopenia (haemolytic anemia, thrombocytopenia, neutropenia); recurrent infections	c.735ofG_p.A247Qfs*46<br>in FASLG gene	normal	-
RF12	dermatitis; enteropathy; hypergammaglobulinemia (IgA)	-	nd	nd

RF13	atopic dermatitis up to 2-3 years of age; punctate lesions with subsequent pus formation, accompanied by itching and scratching lesions; hypergammaglobulinemia (IgE)	nd	RBE ↑; Switched memory B cells ↓	-
RF14	eczema; celiac disease; autoimmune neutropenia; autoimmune thrombocytopenia	-	DNTαβ ↑	-
RF15	multiple lymphadenopathies; autoimmune haemolytic anemia; autoimmune neutropenia; autoimmune thrombocytopenia	-	DNTαβ ↑	-
RF16	arthritis; autoimmune haemolytic anemia; autoimmune thrombocytopenia; lymphoproliferation defect; splenomegaly (spleen removed + paternal familiarity for splenic lymphoma)	-	B lymphocytes ↓; Switched memory B lymphocytes ↑; T lymphocytes CD8+ ↑; RTE ↓	pSTAT3 ↑
RF17	hepatomegaly (linked to HLA) with increased liver enzymes; autoimmune cytopenia; lymphopenia; neonatal hypotonia; persistent increase in inflammation indices; Herpes virus infections; autoimmune thrombocytopenia; mild autoimmune neutropenia	-	B lymphocytes ↑; RBE ↓; T lymphocytes ↓; RTE ↓	FoxP3 ↓
RF18	autoimmune enteropathy (onset at 2 months of life with vomiting, diarrhea, fever and shock); trilinear autoimmune cytopenia (anemia, thrombocytopenia, neutropenia) hypogammaglobulinemia (IgG, IgM)	-	normal	-
RF19	early onset IBD; autoimmune cytopenia; lymphopenia; splenomegaly; unexplained fever	-	B lymphocytes ↓; T lymphocytes CD8+ ↑; RTE ↓; RBE ↑	nd
RF20	autoimmune cytopenia; arthritis; mild splenomegaly; unexplained fever; recurrent infections	-	normal	-
RF21	vitiligo; autoimmune enteropathy; autoimmune thyroiditis (familiarity on the part of grandmother together with splenomegaly)	-	DNTαβ ↑	-
RF22		c.A1721C_p.N574T	normal	STAT1↑

	recurrent oral candidiasis; aphthous stomatitis (RAS); autoimmune thyroiditis, autoimmune gastritis; hemolytic anemia	in STAT1 gene		
RF23	autoimmune thyroiditis; autoimmune gastritis; alopecia; polyarthritis; hypogammaglobulinemia; anemia	c.T310Cp.C104R in TNFRSF13B gene	Switched memory B cells↓	nd
RF24	atrophic gastritis; vitiligo	-	normal	nd
RF25	recurrent fevers; autoimmune thrombocytopenia; autoimmune hemolytic anemia; leukopenia; recurrent infections; severe hypogammaglobulinemia	c.G160A p.A54T in CTLA4 gene	B Lymphocytes ↓; RTE↓	pS6↓
RF26	ulcerative colitis; vitiligo; hepatosplenomegaly; polyarthritis; anemia; hypereosinophilia	-	normal	nd
RF27	vitiligo; scleroderma; mild hypogammaglobulinemia (IgG)	-	normal	nd
RF28	autoimmune thrombocytopenia; severe hypogammaglobulinemia; chronic enteropathy; insulin-dependent type 1 diabetes mellitus; autoimmune thyroiditis; frequent infections	c.C223T p.R75W in CTLA4 gene	B lymphocytes↓; RTE↓	CTLA4↓; pS6↓
RF29	pericarditis; hypereosinophilia; atopic dermatitis; alopecia; coeliac disease	-	normal	-
RF30	diarrhea; atopic eczema; leukocytosis; hypereosinophilia; coeliac disease; hypogammaglobulinemia (IgG)	c.748_750ofAAG_p.K250del in FOXP3 gene	T lymphocytes CD8+ ↑	nd

Note: RF, code to anonymize patients with also progressive numbers based on the timing of enrollment; nd, not done; -, not found. T lymphocytes (CD3+); B lymphocytes (CD19+); RTE = Recent Thymic Emigrants (CD3+CD4+CD31+CD45RA+); RBE = Recent Bone marrow Emigrants (CD19+ CD10+ CD38+); Regulatory T cells (CD3+ CD4+ CD25+ CD127-); DNTαβ = double negative T cells with αβ T cell receptor (CD3+ CD4- CD8- TCRαβ+); Switched Memory B cells (CD19+ CD27+ IgD/IgM-); CD8 memory T cells.
